# Prevalence of serological markers for hepatitis and potential associated factors in patients with diabetes mellitus

**DOI:** 10.1590/1518-8345.2774.3085

**Published:** 2018-11-29

**Authors:** Clarissa Cordeiro Alves Arrelias, Fernando Belissimo Rodrigues, Maria Teresa da Costa Gonçalves Torquato, Carla Regina de Souza Teixeira, Flávia Fernanda Luchetti Rodrigues, Maria Lucia Zanetti

**Affiliations:** 1 Universidade de São Paulo, Hospital das Clinicas, Faculdade de Medicina de Ribeirão Preto, Ribeirão Preto, SP, Brazil.; 2 Universidade de São Paulo, Faculdade de Medicina de Ribeirão Preto, Ribeirão Preto, SP, Brazil.; 3 Prefeitura Municipal de Ribeirão Preto, Secretaria Municipal de Saúde, Ribeirão Preto, SP, Brazil.; 4 Universidade de São Paulo, Escola de Enfermagem de Ribeirão Preto, PAHO/WHO Collaborating Centre for Nursing Research Development, Ribeirão Preto, SP, Brazil.

**Keywords:** Diabetes Mellitus, Hepatitis B, Hepatitis C, Immunization Coverage, Liver Diseases, Nursing

## Abstract

**Objective:**

to estimate the prevalence of serological markers for hepatitis B and C in patients with diabetes mellitus and analyze potential associated factors.

**Method:**

a cross-sectional study with 255 patients with diabetes mellitus. Demographic, clinical, and risk behavior factors for hepatitis B and C were selected. The markers HBsAg, Anti-HBc IgG, Anti-HBc IgM, Anti-HBs, and Anti-HCV were investigated. A questionnaire and venous blood collection and inferential statistical analysis were used.

**Results:**

16.8% of the patients had a total reactive Anti-HBc marker, 8.2% an isolated Anti-HBs, and 75% were non-reactive for all hepatitis B markers. No case of reactive HBsAg was found and 3.3% of the patients had a reactive anti-HCV marker. The prevalence of prior hepatitis B virus infection was directly associated with the time of diabetes mellitus and the prevalence of hepatitis C virus infection was not associated with the investigated variables. The prevalence of hepatitis B and C infection in patients with diabetes mellitus was higher when compared to the national, with values of 16.8% and 3.3%, respectively.

**Conclusion:**

the results suggest that patients with diabetes are a population of higher vulnerability to hepatitis B and C, leading to the adoption of preventive measures of their occurrence.

## Introduction

The international literature shows outbreaks of hepatitis B virus (HBV) and hepatitis C virus (HCV) infection in the hospital, outpatient, and long-term care facilities. Infection cases have been shown to be more frequent in patients with diabetes mellitus (DM) than in those without the disease, suggesting that patients with DM are potentially more susceptible to HBV and HCV infection as a result of treatment and control procedures of the disease, in particular, the monitoring of capillary glycemia^1^ ^-^ ^8^.

These outbreaks occur when infection control standards during capillary glycemia monitoring are neglected, such as the sharing of lancet pens, lancets, and glucometers without the proper disinfection process due to the transmission of microorganisms through the blood. HBV and HCV can survive on surfaces such as lancet pens, lancets, and glucometers on average for five to seven days, even in the absence of visible blood. During this period, the virus can cause infection if it reaches the bloodstream of a susceptible person^9^ ^-^ ^10^.

There is evidence that the severity and lethality related to HBV and HCV infection are higher in patients with DM than in those without the disease. Studies show that in patients infected with HBV and HCV, the presence of DM can accelerate the progression of liver disease, lead to cirrhosis, hepatocellular carcinoma, and death^11^ ^-^ ^12^. In addition, HBV and HCV infection may negatively influence the glycemic control of patients with DM, increasing the risk of hyperglycemia^13^ ^-^ ^15^.

However, in Brazil, there is a shortage of studies regarding the behavior of hepatitis B and C in patients with DM. Regarding hepatitis C, four studies were identified in patients with DM^16^ ^-^ ^18^. One of them showed a high prevalence of hepatitis C in patients with type 2 diabetes mellitus (DM2) when compared to blood donors without DM^16^. Another study also found a high prevalence of hepatitis C in patients with DM2^18^. On the other hand, studies did not identify a difference in the prevalence of hepatitis C in patients with and without DM^14^ and cases of hepatitis C in investigated patients with DM2^17^.

A study on the occurrence of hepatitis showed the magnitude of the prevalence of Hepatitis A, B, and C Virus Infections in the Brazilian macro-regions and represented a major step in coping with hepatitis in Brazil^19^. However, the behavior of the disease in individuals with DM and risk factors related to infection in this population is unknown.

Thus, considering the significant increase in the prevalence of DM in the city of Ribeirão Preto, SP, Brazil, from 12.1% in 1997 to 15.1% in 2006, the impact of HBV and HCV infection on morbidity and mortality, aggravated by DM, that patients with DM constitute an increased risk population for hepatitis B and C, this study aimed to estimate the prevalence of serological markers for hepatitis B and C in patients with DM and analyze potential related risk factors. We believe that the proposed study can provide subsidies to know the magnitude of the problem and to advance the production of knowledge about hepatitis B and C and DM. This study may represent the emergence of a new research topic that could lead to other studies, translating into the quality of health information and, therefore, an improvement in the healthcare network.

## Method

This is a cross-sectional study carried out in a secondary health unit in of a city in the State of São Paulo, Brazil. The study population consisted of 314 patients with type 1 and 2 DM, who attended at least one consultation in the period from July to December 2014. All patients of both sexes, aged 18 years or more, with a diagnosis of type 1 and 2 DM registered in the health records, and who attended the medical consultation from July to December 2014 were considered as eligible. Seven patients were excluded due to hearing or cognitive limitations that made it impossible to answer the questions of the instrument, and other 17 due to the difficulty of establishing contact by the researcher. Thus, 290 patients with DM were invited to participate in the study, of whom 35 refused. The main reasons cited for the refusal were the lack of time to answer the questionnaire, lack of interest in participating in the study because they had already participated in other research projects, and unavailability for blood collection. The convenience sample consisted of 255 patients with DM who attended the medical consultation during the data collection period and met the inclusion criteria. This value (n=255) represents 88% of the patients invited to participate in the study, 81% of the study population and 39% of the patients with DM attended in that unit in 2014. The explanatory variables were demographic (sex, age, and schooling) and clinical (DM time, insulin use, capillary glycemia monitoring, medical, surgical, diagnostic, and therapeutic interventions, behaviors and situations of risk for hepatitis B and C), and the outcomes were HBV and HCV infection.

For this study, the researcher elaborated the questionnaire Occurrence of Serological Markers for Hepatitis B and C in Patients with Diabetes Mellitus based on the questionnaire for adolescents and adults used in the National Survey of Prevalence of Hepatitis A, B and C Virus Infections^20^, the researcher’s experience with patients with DM, and an extensive literature review on the subject^7^ ^-^ ^8^ ^,^ ^19^ ^-^ ^23^. The questionnaire was composed of 96 questions subdivided into five parts: Identification (11 questions); Demographic variables (four questions); Clinical variables (51 questions); Behavioral variables (24 questions); and Results of serology tests for hepatitis B and C (six questions).

The data collection instrument was pre-tested with ten patients in order to identify possible adjustments in the sequence of questions, test the approach to the patient, as well as estimate the time of application of the questionnaire. For data collection, the researcher had the collaboration of a student of Scientific Initiation previously qualified in order to standardize it. After the application of the pre-test, the questionnaire was maintained with no need for adjustments regarding its form and content. The ten patients were included in the final study sample. The data collection was carried out from July to December 2014.

Among the 255 patients, 226 attended the unit to collect blood, 19 performed the collection at home, and 10 patients refused to collect blood. Thus, 245 patients performed blood collection. The main reasons for the refusal were the lack of time and the withdrawal from participation in this phase of data collection.

The statistical analysis of the data was performed using the program STATA 11.0 (StataCorp LP, College Station, USA). The description of demographic and clinical data was presented through descriptive statistics, considering all the patients who participated in the study (n=255). The serological analysis for hepatitis B in patients who underwent blood sampling (n=245) enabled to evaluate the presence of the markers HBsAg, Anti-HBc IgG, Anti-HBc IgM, e Anti-HBs. Because the markers HBsAg and Anti-HBc IgM were non-reactive for all patients for analysis and data presentation, the marker total Anti-HBc was considered as equivalent to Anti-HBc IgG. The serological analysis for hepatitis C allowed evaluating the presence of the marker Anti-HCV. A reactive result for this marker was considered an HCV infection. The univariate analysis of possible associations between demographic and clinical variables and HBV and HCV infection was determined by the Pearson-corrected chi-square test or two-tailed Fisher exact test and Wilcoxon test. The project was approved by the Research Ethics Committee under No. CAAE 24638213.2.0000.5393.

## Results

The demographic and clinical characteristics of the 255 (100%) investigated patients are described in Table 1.

**Table 1:** Distribution of patients with DM* according to demographic and clinical variables. Ribeirão Preto, SP, Brazil, 2014

Demographic variables		n	%
Gender			
	Male	85	33.3
	Female	170	66.7
Age			
	Median (p25-p75)	63 (55-71)	
Schooling			
	Illiterate	9	3.5
	Adult literacy	2	0.8
	Incomplete 1st to 4th grade of Elementary School	51	20.0
	Complete 1st to 4th grade of Elementary School	69	27.1
	Incomplete 5th to 8th grade of Elementary School	26	10.2
	Complete 5th to 8th grade of Elementary School	31	12.2
	Incomplete High School	8	3.1
	Complete High School	39	15.3
	Incomplete Higher Education	10	3.9
	Complete Higher Education	10	3.9
Time of DM* (years)			
	Median (p25-p75)	10 (4-20)	
Use de insulin			
	No	105	41.2
	Yes	150	58.8
Monitoring of capillary glycemia			
	No	65	25.5
	Yes	190	74.5

*DM - Diabetes mellitus

Among the 245 (100%) patients who attended the blood collection, 41 (16.8%) presented a marker corresponding to prior infection with a spontaneous cure, 20 (8.2%) presented vaccination seroconversion and, 184 (75%) presented susceptibility to infection. No cases of acute or chronic hepatitis B were found. Therefore, the prevalence of prior HBV infection found in patients with DM was 16.8%.

Table 2 shows the results obtained from the univariate analysis of prior HBV infection according to demographic and clinical variables. The prior infection had a direct association with age (p=0.014) and DM time (p=0.043). No significant association was observed for the other variables.

**Table 2:** Distribution of patients with DM* with and without prior hepatitis B according to demographic and clinical variables of DM*. Ribeirão Preto, SP, Brazil, 2014

Variables		Total Anti-HBc^†^					p
		(-)			(+)		
		n	%		n	%	
	Total	204	83.2		41	16.8	
Gender							
	Male	63	30.9		17	41.5	
	Female	141	69.1		24	58.5	0.187^‡^
Age							
	Median (p25-p75)	62.8 (55.2-69.3)			68.4 (58.9-75.2)		0.014^§^
Schooling							
	Illiterate	6	2.9		3	7.3	
	Adult literacy	1	0.5		1	2.4	
	Incomplete 1st to 4th grade of Elementary School	40	19.6		9	22.0	
	Complete 1st to 4th grade of Elementary School	57	28.0		8	19.5	
	Incomplete 5th to 8th grade of Elementary School	19	9.3		6	14.6	
	Complete 5th to 8th grade of Elementary School	25	12.3		4	9.8	
	Incomplete High School	8	3.9		-	-	
	Complete High School	31	15.2		7	17.1	
	Incomplete Higher Education	8	3.9		2	4.9	
	Complete Higher Education	9	4.4		1	2.4	0.521^II^
Time of DM^*^ (years)							
	Median (p25-p75)	10 (4-19)			12 (10-23)		0.043^II^
Use of insulin							
	No	82	40.2		19	46.3	
	Yes	122	59.8		22	53.7	0.466^‡^
Monitoring of capillary glycemia							
	No	51	25.0		10	24.4	
	Yes	153	75.0		31	75.6	0.934^‡^

*DM - Diabetes mellitus; †Anti-HBc - Antibody (IgM or IgG) against hepatitis B virus core antigen; ‡Pearson-corrected chi-square test; §Wilcoxon test; ||Two-tailed Fisher exact test

Table 3 shows the univariate analysis of prior infection according to variables related to the history of medical, surgical, diagnostic, and therapeutic interventions and situations and behaviors of risk for hepatitis B. The results show an association between prior infection and report of home contact with case of hepatitis B (p=0.001), work as a police officer (p=0.016), and higher number of sexual partners throughout life (p=0.004).

**Table 3:** Distribution of patients with DM* with and without prior hepatitis B according to the history of medical, surgical, diagnostic, and therapeutic interventions and situations and behaviors of risk for hepatitis B. Ribeirão Preto, SP, Brazil, 2014

Variables		Total Anti-HBc^†^					p
		(-)			(+)		
		n	%	n	%		
	Total	204	83.2		41	16.8	
History of interventions^‡^							
	Hospitalization	132	64.7		27	65.8	0.888^§^
	Surgery	161	16.3		33	80.5	0.822^§^
	Blood/derivative transfusion	40	19.6		4	9.8	0.181^II^
	Dental treatment	162	79.4		29	70.7	0.221^§^
	Endoscopy	75	36.8		14	34.2	0.750^§^
	Hemodialysis	3	1.5		-	-	1.000^II^
Situations and behaviors of risk^‡^							
	Home contact with case of hepatitis B	3	1.5		6	14.6	0.001^II^
	Sexual contact with case of hepatitis B	1	0.5		-	-	1.000^II^
	Sharing of sharps	84	41.2		13	31.7	0.258^II^
	Tattoo	8	3.9		1	2.4	1.000^II^
	Piercing	4	2.0		-	-	1.000^II^
	Health professional	19	9.3		4	9.8	1.000^II^
	Work as a police officer	1	0.5		3	7.3	0.016^‡^
	Work as a penitentiary agent/prison officer	1	0.5		-	-	1.000^II^
	Worker collecting household/hospital waste	7	3.4		1	2.4	1.000^II^
	Work as a manicurist/chiropodist/podiatrist	11	5.4		1	2.4	0.696^II^
	Smoked drugs	3	1.5		1	2.4	0.522^II^
	Smelled drugs	2	1.0		1	2.4	0.424^II^
	Condom use	16	7.8		2	4.9	1.000^II^
	Sexually transmitted disease	30	14.7		8	19.5	0.438^II^
	Number of sexual partners throughout life Median (p25-p75)	1 (1-3.5)			3 (1-10)		0.004^¶^
Frequency of alcohol consumption (last three months)							
	None	153	75.0		29	70.7	
	Once a month	19	9.3		4	9.8	
	Two to three times a month	15	7.4		3	7.3	
	One to two days a week	13	6.4		2	4.9	
	Three to four days a week	1	0.5		3	7.3	
	Almost everyday	2	1.0		-	-	
	Every day	1	0.5		-	-	0.202^‡^

*DM - Diabetes mellitus; †Anti-HBc - Antibody (IgM or IgG) against hepatitis B virus core antigen; ‡Non-mutually exclusive categories; §Pearson-corrected chi-square test; ||Two-tailed Fisher exact test; ¶Wilcoxon test

The explanatory variables included in the logistic regression analysis were those that showed a possible association with the outcome (p≤0.20). Among the variables included in the model, disease duration remained directly associated with the prior infection after the multivariate analysis, in which the DM time increases the risk of hepatitis B in approximately 4% each year of diagnosis of the disease. In addition, the work as a police officer was associated with infection (Table 4).

**Table 4:** − Logistic regression model for prior hepatitis B. Ribeirão Preto, SP, Brazil, 2014

Variables*	OR^†^ (95% CI^‡^)	p	Standard error
Female	0.74 (0.32-1.71)	0.487	0.31
Age	1.02 (0.99-1.06)	0.143	0.01
Time of DM^§^	1.04 (1.00-1.08)	0.024	0.01
Home contact hepatitis B	0.97 (0.85-1.10)	0.658	0.06
Work as a police officer	13.82 (1.27-149.94)	0.031	16.81
Sexual partners throughout life	1.00 (0.99-1.00)	0.927	0.001
Alcohol consumption	1.04 (0.75-1.44)	0.806	0.17
Blood/derivative transfusion	0.55 (0.17-1.72)	0.309	0.32

*Those that showed p≤0.20 in the univariate analysis were included. Each variable was adjusted for the other seven; †OR - Odds ratio; ‡CI - Confidence interval; §DM - Diabetes mellitus

Among the 245 investigated patients, 8 (3.3%) presented a reactive anti-HCV marker. Therefore, the prevalence of HCV infection found in patients with DM was 3.3%. No significant association was found between the investigated demographic and clinical variables and HCV infection.

## Discussion

When comparing the obtained results with the population-based survey conducted in Brazil, the prevalence of HBV exposure (16.8%) was higher than the national prevalence (11.6%) in the general population from 20 to 69 years. Regarding the prevalence of acute or chronic infection, the prevalence found was lower than the national prevalence, with a value of 0.6%^19^. This result suggests that the prevalence of HBV exposure is higher in individuals with DM when compared to those without the disease^5^.

The prevalence of prior cured infection and vaccine immunity marker were higher than in Spain^24^. On the other hand, studies carried out in Poland and Turkey showed two-fold higher values^25^ ^-^ ^27^. Other studies also found a higher prevalence^16^ ^,^ ^25^ ^-^ ^29^.

The association of exposure to HBV and a longer time of DM can be interpreted as a cumulative risk of exposure to the virus probably attributed to the disease management since DM does not progress to hepatitis B or C. The association of exposure to HBV and a longer time of DM were reported in Poland^26^, Turkey^28^, and Nigeria^30^. On the other hand, a study carried out in Italy found no association of infection and time of DM^27^.

In accordance with another study^27^, the present study also did not find an association of HBV infection with demographic variables, variables related to insulin use, monitoring of capillary glycemia, and history of medical, surgical, diagnostic, and therapeutic interventions. In addition, the majority of the investigated patients monitored capillary glycemia at home and outbreaks of HBV infection reported in the literature were in institutionalized patients and health services undergoing assisted monitoring of capillary glycemia without proper infection control practices^1^ ^-^ ^2^ ^,^ ^4^ ^,^ ^6^.

The prevalence of HCV infection was 3.3% higher than the national prevalence for the general population from 20 to 69 years old, which was 1.6%^24^ ^,^ ^27^, as well as national studies with specific populations such as the deaf, military males, and workers collecting household waste^31^ ^-^ ^33^. On the other hand, a study that investigated the prevalence of HCV infection in elderly patients in southern Brazil found a prevalence of 2.2%^34^.

The prevalence of hepatitis C in patients investigated in our study was also higher than that found in three national studies with patients with DM^16^ ^-^ ^17^. The difference in the observed prevalence can be attributed to the composition of the sample regarding the age group. An old age is considered a risk factor for exposure to HCV infection^20^ ^-^ ^21^. The time of DM found in these studies was also lower than that found in our study, which may also justify the difference in the observed prevalence.

On the other hand, a study carried out in southern Brazil showed that the prevalence was four times higher in patients with DM2 undergoing outpatient care^18^. The time of DM of the investigated outpatients is higher when compared to those of our study, which may have contributed to a higher prevalence of infection.

International studies investigating the prevalence of HCV exposure in patients with DM in an outpatient clinic or hospital found a lower^7^ ^,^ ^24^ ^,^ ^30^, similar^23^ ^,^ ^28^, and higher^8^ ^,^ ^22^ ^,^ ^25^ ^,^ ^27^ ^,^ ^29^ prevalence in relation to our results.

Since the 1990s studies have shown a higher prevalence of hepatitis C in patients with DM when compared to those without this disease^7^ ^-^ ^8^ ^,^ ^16^ ^,^ ^23^ ^,^ ^27^. When comparing the prevalence of hepatitis C in patients with DM found in our study (3.3%) and the prevalence observed in the general Brazilian population (1.4%)^19^, we also observed a higher prevalence of infection in patients with DM.

However, in our study, although we found a prevalence of HCV infection higher than that of the Brazilian population, we did not observe an association of HCV infection with demographic variables, variables related to insulin use, monitoring of capillary glycemia, and history of medical, surgical, diagnostic, and therapeutic interventions, which is in agreement with national studies^16^ ^-^ ^18^.

Other studies reported in the international literature found as variables associated with infection only recognized risk factors for hepatitis C such as the history of blood transfusion, sharing of sharps, multiple sexual partners, and changes in liver enzyme levels^16^ ^,^ ^18^ ^,^ ^23^ ^,^ ^25^ ^,^ ^29^.

A study carried out in France found a significant difference in the prevalence of HCV infection in patients with (3.1%) and without DM (0.04%). However, the hypothesis that the type of treatment for DM, previous hospitalizations, and lancet use pen for monitoring the capillary glycemia are associated with HCV infection in patients with DM has not been confirmed^35^.

These results lead to the assumption that HCV infection may present as a risk factor for the development of DM, as investigated in other studies^36^ ^-^ ^37^. Studies have shown that HCV infection is followed by defects in the insulin-signaling pathway in the liver, which may contribute to insulin resistance and DM^37^. However, HCV-induced insulin resistance mechanisms are still partially understood^14^ ^,^ ^38^. Another study shows that liver inflammation is a possible risk factor for pre-diabetes in the context of HCV infection^39^.

In summary, when considering the higher prevalence of HBV exposure and its relation to the time of DM, it is suggested to deepen new investigations related to diabetes management that may contribute to HBV infection. The absence of association of HCV infection with the studied variables can be attributed to the relatively low number of infected individuals. This research is a pioneer in Brazil and offers subsidies for comparisons with future studies and advances in the knowledge of the subject.

This study offers subsidies to know the magnitude of the problem and advance the production of knowledge about hepatitis B and C and DM. The study can generate new research themes, translating into the quality of health information and, therefore, qualification of nursing care.

## Conclusion

The prevalence of HBV infection in patients with DM was 16.8%, which is higher than the national level and was directly associated with the time of DM. No cases of acute or chronic hepatitis B were found. The prevalence of HCV infection was 3.3%, which is higher than the national level and had no association with the investigated demographic and clinical variables. Further studies need to be developed to investigate these issues and deepen the knowledge of the relationship between hepatitis C and DM in the national population aiming at the timely adoption of preventive measures.
